# The First Case of Kleefstra Syndrome in a Rwandan Patient with Global Developmental Delay

**DOI:** 10.3390/genes17040429

**Published:** 2026-04-07

**Authors:** Norbert Dukuze, Janvier Hitayezu, Jeanne Primitive Uyisenga, Esther Uwibambe, Jean Hubert Caberg, Vinciane Dideberg, Vincent Bours, Abdullateef Isiaka Alagbonsi, Leon Mutesa, Annette Uwineza

**Affiliations:** 1 Center for Human Genetics, School of Medicine and Pharmacy, College of Medicine and Health Sciences, University of Rwanda, KG 11 Ave Gasabo, Kigali P.O. Box 3286, Rwanda; norbertduk123@gmail.com (N.D.); uwaesther04@gmail.com (E.U.); annetteuwineza@gmail.com (A.U.); 2 Department of Biochemistry, Molecular Biology and Genetics, School of Medicine and Pharmacy, College of Medicine and Health Sciences, University of Rwanda, Kigali P.O. Box 4285, Rwanda; 3 Center for Human Genetics, Centre Hospitalier Universitaire Sart-Tilman, University of Liege, Sart Tilman, 4000 Liège, Belgium; jh.caberg@chuliege.be (J.H.C.); vinciane.dideberg@chuliege.be (V.D.); vbours@uliege.be (V.B.); 4 Department of Pediatrics, University Teaching Hospital of Kigali (CHUK), Kigali P.O. Box 655, Rwanda; jhitayezu@gmail.com; 5 Department of Biology, College of Science and Technology, University of Rwanda, Kigali P.O. Box 3900, Rwanda; jusenga18@gmail.com; 6 Department of Physiology, School of Medicine and Pharmacy, College of Medicine and Health Sciences, University of Rwanda, Kigali P.O. Box 3286, Rwanda; a.i.alagbonsi@ur.ac.rw

**Keywords:** Kleefstra syndrome, *EHMT1*, exome sequencing, neuro-developmental disorders, Rwanda

## Abstract

**Background:** Kleefstra syndrome (KS) is a rare neurodevelopmental disorder caused by haploinsufficiency of *EHMT1*; it is characterized by global developmental delay, intellectual disability, hypotonia, distinctive facial features, and variable congenital anomalies. Autistic features, behavioral abnormalities and severe speech impairment are frequently reported. However, molecularly confirmed cases of KS from Africa remain extremely limited, largely due to restricted access to genomic diagnostic infrastructures. **Methods:** We describe a 15-month-old patient from Rwanda presenting with neonatal hypotonia, global developmental delay, short stature, and characteristic dysmorphic facial features. Comprehensive clinical evaluation was performed, followed by trio-based exome sequencing to identify the underlying genetic cause of this neurodevelopmental disorder. **Results:** Exome sequencing identified a de novo heterozygous frameshift variant in *EHMT1* (NM_024757.5: c.2871dup; p. Phe958Leufs*219), confirming the diagnosis of KS. **Conclusions:** This report presents the first molecularly confirmed case of KS in Rwanda. It highlights additional clinical features like bilateral 5th toe clinodactyly, short stature and absence of obesity in KS. There is a need to further delineate the study of *EHMT1* and investigate the natural history of KS across different populations for optimal patient management and to reduce diagnostic odyssey. The diagnostic utility of exome sequencing for neurodevelopmental disorders needs to be strengthened, with strong emphasis on expanding genomic medicine to help diagnose rare diseases in resource-limited settings.

## 1. Introduction

KS (OMIM 610253) is a rare neurodevelopmental disorder (NDD) caused by the deletion of the chromosome 9q34.3 genomic sequence or loss of function of euchromatin histone methyltransferase 1 (*EHMT1*) with OMIM number 607001 [1]. *EHMT1* encodes a histone methyltransferase that modulates gene expression by altering chromatin architecture and by interacting with additional transcriptional regulators [2]. KS is a multisystem disorder involving abnormalities of the neurological, cardiovascular, musculoskeletal, gastrointestinal, renal, and genitourinary systems, with key clinical features including developmental delay or intellectual disability, autism-like characteristics, childhood hypotonia, and distinct facial features [3]. To date, KS is estimated to affect between 1:25,000 and 1:35,000 individuals globally [4]. Currently, cases of KS have only been reported in South Africa, Egypt and Morocco among the African population, while most other reports are from non-African populations [5]. Bouman et al. (2024) used the Genetics of Intellectual Disability and Autism Spectrum Disorders data to identify cases from South Africa and Egypt, whereas the Moroccan cases were reported from data of the Radboud University Medical Center biobank for Genetics and Rare Diseases, Netherlands [6]. To the best of our knowledge, no case of KS has been reported in the East African region or Rwanda in particular, likely due to limited availability of molecular diagnostic testing infrastructure. In this study, we report the clinical phenotype of the first molecularly confirmed case of KS. The condition presents with a clinically recognizable phenotype including developmental milestone delay and behavioral features. Males and females are equally affected [7]. There is mounting evidence that the etiology of global developmental delay and cognitive dysfunction is significantly influenced by the epigenetic change in the chromatin structure in neurons, which controls *EHMT1* transcription [8]. A cost-effective approach to improve the quality of genetic diagnosis of neurodevelopmental disorders like Kleefstra syndrome is the examination of structural variants, such as copy number variations or single-nucleotide variants, by using exome sequencing technologies [9]. The *EHMT1* expression pattern indicates how it plays a role in central nervous system development and function. This aligns with the severe intellectual disability and behavioral issues observed in patients with a deletion of one copy of the gene [10]. *EHMT1* is also a gene implicated in brain development, and it is increasingly recognized for its role in chromatin remodeling. The full profile of clinical presentations and genotype–phenotype correlations of KS is not fully understood [11]. Given the widespread interest in the role of *EHMT1* in cell biology and cancers, the increase in *EHMT1* variants found in people with KS enables more precise characterization of both molecular and phenotypic aspects as well as a more comprehensive understanding of *EHMT1* functions [12]. Genetic testing in sub-Saharan countries remains limited, with only three geneticists serving a population of 14 million in Rwanda. Clinical diagnostics rely mainly on karyotyping, restricting detection of conditions like KS and other rare genetic conditions [13]. This case report highlights a novel clinical presentation and expands the spectrum of *EHM1* variants responsible for KS.

## 2. Materials and Methods

The study was approved by the Institutional Review Board of the College of Medicine and Health Sciences at the University of Rwanda (500/CMHS IRB/2024) and Kigali University Teaching Hospital. The patient was evaluated by a trained medical geneticist in a genetic clinic. The diagnosis of global developmental delay was based on evaluating the patient’s development quotient (DQ) by using the Gesell Development Diagnosis Scale (for children under five years old). A thorough physical exam and laboratory investigation was also performed by a trained geneticist.

### 2.1. Case Presentation

**Figure 1:**
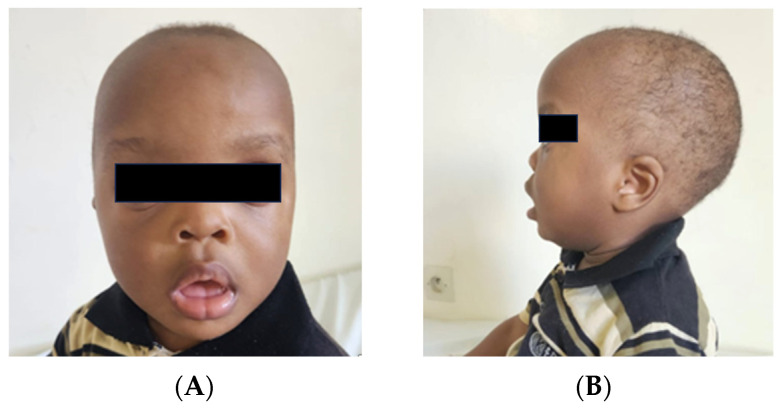
(**A**) Frontal facial view showing a wide forehead, hypertelorism, a depressed nasal bridge, anteverted nares, a tented upper lip, an everted lower lip, and midface hypoplasia. (**B**) Profile view highlighting midface hypoplasia and facial gestalt consistent with KS.

The patient is a 15-month-old male, who was referred to the genetic clinic of the University Teaching Hospital of Kigali for global developmental delay and dysmorphic facial features. The patient is the third child of healthy nonconsanguineous parents and there is no family history of intellectual disability or congenital malformations. He was born to a 39-year-old gravida 6 para 6 mother, by cesarian section at term with a birth weight of 2.785 kg. The patient presented hypotonia during the neonatal period. He underwent cardiac evaluation and the echocardiogram was normal. On physical examination (at 15 months), his weight was (9.8 kg = 25th percentile) and occipitofrontal circumference (OFC) was (46 cm = 50th percentile), with a short stature observed (height 69 cm below 3rd percentile), using the WHO growth charts. Dysmorphic facial features consisted of low-set ears, a wide forehead, hypertelorism, a depressed nasal bridge, anteverted nares, a tented upper lip and everted lower lip, midface hypoplasia (Figure 1), small teeth and bilateral 5th toe clinodactyly. He had axial hypotonia. At the age of 24 months, developmental reassessment confirmed persistent global developmental delay, with attainment of crawling only with the aid of physiotherapy and continued absence of independent walking and expressive speech. His weight was 11.74 kg (=50th percentile), with an OFC of (48 cm = 50th percentile). In contrast, his short stature remained markedly clear, with a height 74 cm below the 3rd percentile. The karyotype was normal (46, XY).

### 2.2. Trio Whole Exome Sequencing

Trio exome sequencing is a genomic test in which the protein-coding region of the proband and both biological parents are sequenced and analyzed; up to 30–40% of severe neurodevelopmental disorders result from de novo mutations, and trio-WES is the most effective method to capture them accurately [14]. Peripheral blood was collected from both the patient and the parents. The WES was performed at the University of Liège Centre for Human Genetics. Extraction of DNA from blood samples was performed manually using a QIA amp DNA mini kit (Qiagen) in accordance with the manufacturer’s protocol. The purity and concentration of extracted DNA was assessed using the Nanodrop 1000 UV-spectrophotometer. Subsequently, 30 µL of the normalized lysis product was utilized for library preparation with the Illumina DNA PCR-Free Prep, Tagmentation library preparation kit and IDT^®^ for Illumina^®^ DNA/RNA Unique Dual Indexes Set A, Tagmentation (96 Indexes). The preparation of the exome library was carried out using “Comprehensive Exome” solution from the firm Twist Bioscience; this modified exome targets more than 99% of protein-coding genes (+/−36.8 Mb of regions covered). The process consists of the enzymatic fragmentation of genomic DNA followed by the preparation of this fragmented gDNA before capturing the regions of interest; then, there is the enrichment of the captured regions and high-throughput sequencing of the libraries created on an Illumina NovaSeq 6000 sequencing Technologies with a Flow Cell in 2 × 100 Paired-End sequencing. Sequencing data generated from the patient by NovaSeq were presented in the form of standard fastq.gz files, including a list of inferred variants compared to the reference genome, and a bam file including the mapped reads. All data were processed by bioinformaticians and processed using the Humanomics pipeline. The vcf.gz files including inferred variants from exome sequencing were analyzed via the CE-IVD Agilent Alissa Interpret platform, as well as bam files including mapped reads which allow for visualization of aligned sequences in the IGV genomic data visualization software [15]. Sanger confirmation was not performed for this variant due to the fact that the sequencing was performed in a certified diagnostic genetic laboratory (BELAC certification, ISO 15189) [1]. We validated our bioinformatic WES pipeline using reference genomes from the Ga4GH consortium (GIAB HG001 and HG002 DNA). This consortium defined regions of high confidence for these reference genomes. We certified our workflow by using these regions of high confidence with excellent performance metrics, and every variant that fell out of these high-confidence regions was verified by Sanger. The *EHMT1* heterozygous reported variant was located in a high-confidence region, supported by 251 reads, and it had an allelic fraction of 50.2%. The proband was analyzed alongside both parents, and the coverage of the parents for this region was also higher than 250×, with a relatedness calculated by Somalier, which confirmed the relation to both the mother and father (relatedness of 0.5).

## 3. Results

### 3.1. Whole Exome Sequencing Results

The proband trio-WES identified a variant—NM_024757.5: c.2871dup, p. (Phe958Leufs*219)—in a heterozygous state in *EHMT1*. The variant was absent from control population databases, including the Exome Sequencing Project, 1000 Genomes Project, ExAC, and gnomAD. The duplication results in a frameshift and introduces a premature stop codon 219 amino acids downstream, in a gene for which loss of function is an established pathogenic mechanism (gnomAD pLI = 1). Trio analysis confirmed the variant as de novo. Given the phenotypic consistency with the associated gene, a ClinGen score of 0.5 was used to weight the PS2 score applied to de novo variants. This was based on two main aspects: the phenotype of the patient was consistent with the gene being non-highly specific, of high genetic heterogeneity, and a de novo variant after confirming the parental relationships; the variant occurred in exon 20 of 27 and was therefore subject to nonsense-mediated decay (NMD) and loss of function, and so it was classified as PVS1 [16]. The results were analyzed via intellectual disability and an epilepsy gene panel, the variant was classified as PM2-supporting, and it was absent in the controlled population, including in the Exome Sequencing Project, 1000 Genomes Project, and Exome Aggregation Consortium, GnomAD. It was a PS2-supporting de novo phenotype consistent with the gene but not highly specific and there was high genetic heterogeneity. Given the fact that the variant was found by using a gene panel, robust internal validation studies were conducted at the University of Liege’s Center for Human Genetics’ laboratories. With a high read depth (>100×) and strong bioinformatics pipelines, together with a good variant allele fraction, no other validation process was needed like Sanger sequencing, as the laboratory is a certified diagnostic genetic facility (BELAC certification, ISO 15189). We validated our bioinformatic WES pipeline using reference genomes from the Ga4GH consortium (GIAB HG001 and HG002 DNA) [15]. The ClinGen sequence variant interpretation framework assigns points to de novo variants based on phenotype specificity and gene–disease relationship strength. The reported case’s phenotype fitted KS but was not specific to the *EHMT1* variant. There were no other contiguous genes affected in the 9q34.3 region as reported by exome sequencing results, and no copy number variants were detected. The identified variant was NM_024757.5: c.2871dup, p. (Phe958Leufs*219), a heterozygous state of *EHMT1* (OMIM*607001; OMIM#610253 Kleefstra syndrome 1). The heterozygous frameshift variant in *EHMT1* fitted ACMG criteria (PVS1, PM2-supporting, and PS2-supporting), and it was categorized as pathogenic since it introduces an early stop codon that causes loss of function. It was absent from population databases and occurred de novo in a compatible phenotype [16]. The clinical presentations and identified genetic marker of the patient are shown in Table 1 and The read alignment and variant visualization supporting the detected variant are provided in the Supplementary Materials (Supplementary Figure S1), generated using Integrative Genomics Viewer (IGV).

### 3.2. Imaging Analysis

Brain and spine MRI of the patient did not reveal any abnormalities.

## 4. Discussion

The identification of pathogenic variants in *EHMT1* in our patient highlights the expanding genetic and phenotypic spectrum of KS in African populations where genomic data remain significantly under-represented [17]. Specifically, the identified variant—NM_024757.5: c.2871dup, p. (Phe958Leufs*219)—has not been previously reported and further supports loss of function as the primary pathogenic mechanism underlying KS. Our patient’s phenotype aligns with the characteristic clinical spectrum documented in published KS cohorts [3]. The patient presented early with neonatal hypotonia, severe global developmental delay, and absent expressive speech by the age of 3 years, findings consistently reported as cardinal features in major case series and review [11]. However, additional clinical features frequently reported in other KS patients, such as behavioral abnormalities, autism spectrum disorder, sleep disturbances, epilepsy, congenital heart defect and urogenital malformations [18], were absent in our patient at the time of evaluation. This observation is consistent with prior reports demonstrating that the KS phenotype is age-dependent and may evolve over time, highlighting the importance of prospective clinical follow-up in affected individuals. The patient’s short stature (height < 3rd percentile on WHO charts) with relatively preserved weight and head circumference at 15 months aligns with prior observations that growth can be variably affected across individuals with KS [6]. Identification of KS in African patients remains particularly challenging because diagnosis based solely on clinical features is difficult, as the characteristic facial gestalt may be subtle, age-dependent, or underrecognized in individuals of African ancestry due to limited normative phenotypic references and the marked under-representation of African patients in published KS cohorts. This case highlights how reliance on clinical features alone can result in delayed or missed diagnoses, reinforcing the critical importance of genomic testing for achieving diagnostic equity in low- and middle-income countries (LMICs). Studies indicate that neurodevelopmental disorders including KS affect up to 10–17% of children, yet most patients in sub-Saharan Africa, including Rwanda, do not undergo advanced genetic testing, with <25% of studies using next-generation sequencing [19]. Policy efforts should therefore emphasize equitable access, local capacity building, and incorporation of genomics into routine patient clinical care [20]. The reported case reinforces the core KS phenotype, namely, hypotonia, severe global developmental delay, and absent speech, consistent with large cohorts [21]. However, the absence of obesity and seizures and the presence of short stature together with bilateral 5th toe clinodactyly highlight phenotypic variability, as also observed in recent genotype–phenotype studies [12]. This case highlights underappreciated facial gestalt distinctions in African ancestry as compared to European cohorts [22]. Due to restricted genetic access in sub-Saharan countries, Rwanda in particular continues to face significant diagnostic challenges that frequently results in delayed or missed diagnosis of neurodevelopmental disorders. This study shows how important trio-WES is for resolving clinical uncertainty and lowering diagnostic disparities in resource-limited settings [23]. Appendix A is first referenced here and summarizes the key findings of this study; it illustrates the clinical presentation alongside the identification of *EHMT1* variant through trio-whole exome sequencing in KS.

## 5. Conclusions

KS shares clinical presentations with other neurodevelopmental disorders and it is challenging diagnosis. In the case of clinical suspicion in a patient with psychomotor developmental delay, *EHM1* should be systematically evaluated. In the future, better delineation of the natural history of KS is required to allow for adequate phenotype recognition. The investigation of *EHMT1*, a key regulator in the epigenetic process, could provide new insight into disease mechanisms and therapeutic options. Therefore, it is paramount to emphasize the critical importance of integrating trio exome sequencing into the diagnostic evaluation of neurodevelopmental disorders even in under-represented populations.

## Figures and Tables

**Table 1:** Clinical presentations of KS in Rwandan patient compared to other patients.

Comparison of Reported Kleefstra Syndrome Clinical Features vs. Rwandan Patient with Kleefstra Syndrome			
System/Feature	Typical Kleefstra Syndrome Symptoms	Rwandan Patient Symptoms	Novel/Not Commonly Emphasized
Variant	EHMT1 haploinsufficiency (deletions or loss of function variants)	EHMT1: c.2871dup; p. Phe958Leufs*219 (de novo frameshift)	Novel variant (previously unreported)
Inheritance	Autosomal dominant (mostly de novo)	De novo confirmed by trio-WES	—
Growth	Obesity (later childhood), variable growth	Short stature (<3rd percentile), normal weight percentile	Early short stature without obesity
Head size	Microcephaly	Normal OFC (50th percentile)	Normal head circumference despite GDD
Hypotonia	Common (early infancy)	Neonatal hypotonia, persistent axial hypotonia	—
Global developmental delay	Severe	Severe GDD, delayed motor milestones	—
Speech	Severe speech delay or absent speech	No expressive speech at 24 months	—
Face (general)	Coarse facies, flat face	Flat face	Absence of coarse facies
Forehead	Not consistently described	Wide forehead	Possibly underreported feature
Eyes	Hypertelorism, up-slanting palpebral fissures	Hypertelorism	—
Ears	Malformed ears and hearing loss	Low-set ears	—
Nose	Anteverted nares	Anteverted nares + depressed nasal bridge	Depressed nasal bridge emphasized
Midface	Midface hypoplasia	Midface hypoplasia and short neck	—
Mouth	Everted lower lip	Tented upper lip + everted lower lip	Tented upper lip (less commonly highlighted)
Dentition	Not consistently reported	Small teeth	Rarely reported feature
Extremities (hands/feet)	Brachydactyly, single palmar crease	Bilateral 5th toe clinodactyly	Toe clinodactyly (less typical than hand anomalies)
Cardiac defects	Cono-truncal defects	Normal echocardiogram	Absence of CHD
Neurologic (seizures)	Seizures	No seizures reported	Absence at early age
Behavioral/psychiatric	Autism, aggression, OCD, sleep disorders	Not observed at 15–24 months	—
Brain imaging	May show abnormalities	Normal brain MRI	Normal imaging
Other anomalies	Urogenital, renal anomalies possible	None reported	Absence of systemic anomalies

## Data Availability

The original contributions presented in this study are included in the article/Supplementary Materials. Further inquiries can be directed to the corresponding author.

## Supplementary Materials

The following supporting information can be downloaded at: https://www.mdpi.com/article/10.3390/genes17040429/s1, Figure S1. IGV for Patient. NM_024757.5: c.2871dup, p. (Phe958Leufs*219) in heterozygote status of *EHMT1* (OMIM*607001; OMIM#610253 Kleefstra syndrome 1).

## Abbreviations

The following abbreviations are used in this manuscript:

WES	Whole Exome Sequencing
ACMG	American College of Medical Genetics
KS	Kleefstra Syndrome
CHUK	Kigali University Teaching Hospital
CNV	Copy Number Variant
SNV	Single-Nucleotide Variant
IRB	Institute Review Board
LMIC	Low- and Middle-Income Country
IGV	Integrative Genomics Viewer
DNA	Deoxyribose Nucleic Acid

## Appendix A

The graphical figure below illustrates the clinical to molecular diagnostic pathway of the first case of KS in Rwanda and, to the best of our knowledge, in East Africa. It starts with neonatal presentation that includes dysmorphic features including a depressed nasal bridge, a wide forehead, low-set ears, midface hypoplasia and marked hypotonia, along with global developmental delay. It emphasizes how trio whole exome sequencing was used as a decisive diagnostic technique that revealed a pathogenic frameshift variant in *EHMT1* (c.2871dup; p. Phe958Leufs*219) in the proband, while the parents’ results were normal. The variant was classified as Class V according to the American College of Medical Genetics criteria. The final segment of the figure highlights the strong need for a multidisciplinary team approach in the management of global developmental delay and the role of expanding access to advanced genomic testing to improve early diagnosis and provide individualized care to patients with neurodevelopmental disorders.
