# Supplementary material for: The First Case of Kleefstra Syndrome in a Rwandan Patient with Global Developmental Delay
Source: Genes (Basel). 2026 Apr 7;17(4):429. doi: 10.3390/genes17040429 (PMC13115583; doi:10.3390/genes17040429)
Supplement: Supplementary file 1 [file genes-17-00429-s001.zip › genes-4192380-supplementary.pdf]

Supplementary Material:

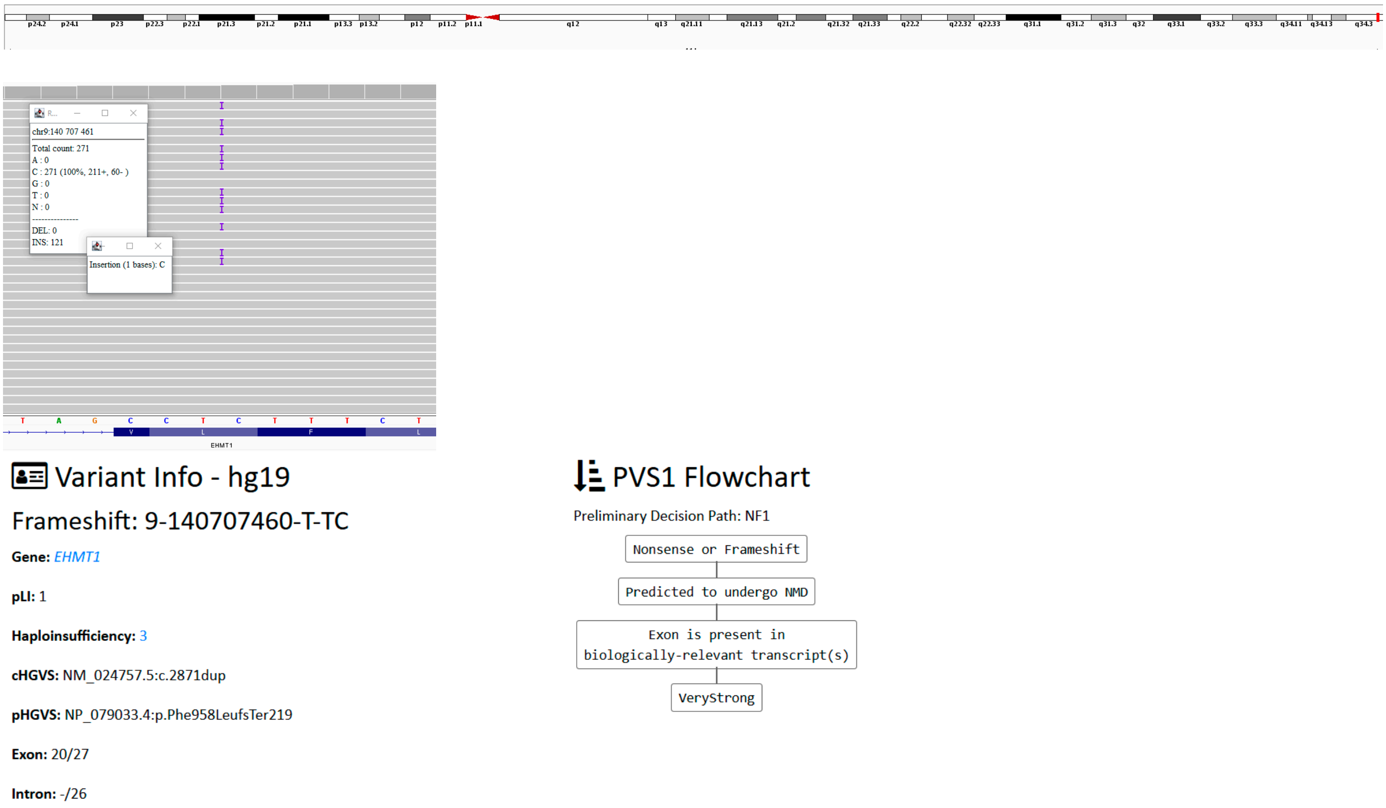

Figure S1. IGV for Patient. NM_024757.5: c.2871dup, p. (Phe958Leufs*219) in heterozygote status of *EHMT1* (OMIM*607001; OMIM#610253 Kleefstra syndrome 1).
